# Primary spinal meningeal melanoma with intramedullary and intradural extramedullary components—a case report

**DOI:** 10.1093/bjrcr/uaaf020

**Published:** 2025-03-25

**Authors:** Robert H Bardsley, Jasmine Kimber, Kassie McCullagh

**Affiliations:** Department of Surgery, University of North Carolina at Chapel Hill, Chapel Hill, NC 27599, United States; University of North Carolina School of Medicine, Chapel Hill, NC 27599, United States; Department of Radiology, University of North Carolina at Chapel Hill, Chapel Hill, NC 27599, United States

**Keywords:** melanoma, primary melanoma of meninges, primary intradural extramedullary melanoma, primary intramedullary melanoma

## Abstract

Primary melanomas of the spinal meninges are exceedingly rare. While both intramedullary and extramedullary spinal melanomas have been reported, to the best of our knowledge, this is the first noted case of primary spinal melanoma that has both intramedullary and intradural extramedullary components. We present a case of a 61-year-old female presenting with a 1-year history of lower back pain, bilateral lower extremity pain, and perceived weakness of left foot. Magnetic resonance imaging of the thoracic spine suggested intramedullary and intradural extramedullary mass at levels T8-T12. A T7-T12 laminectomy with resection of the spinal cord mass revealed a pathological diagnosis of primary meningeal melanoma. This case highlights the complexity of diagnosing spinal melanomas, which often mimic more common spinal tumours such as ependymomas, astrocytomas, metastasis, or lymphoma. Often meningeal melanomas require extensive imaging and clinical evaluation to exclude other sites of potential primary melanoma. This case adds to the sparse literature by documenting a rare manifestation and could provide valuable insights into the diagnosis and management of similar cases.

## Introduction

Melanoma, a malignant neoplasm that originates from melanocytes or predecessor melanocytes, can manifest anywhere in the body, including the central nervous system (CNS).^1^ Primary CNS melanomas are particularly rare and account for about 1% of all melanomas and are thought to originate from melanoblasts associated with the pial sheaths of vascular bundles or from neural crest cells during embryogenesis.^1^^,^^2^ The incidence of primary spinal melanomas is even less frequent and can be intradural extramedullary (∼62%) or intramedullary (∼38%) in location.^1^^,^^2^ Sparse examples in the literature exist for spinal melanomas.^2^ We present a case of a 61-year-old female evaluated for gradually worsening low back pain, with subsequent MRI showing a thoracic spinal mass with intramedullary and extramedullary components and diagnosed as a primary meningeal melanoma after resection. To the best of our knowledge, this is the first reported case of primary spinal melanoma with both intramedullary and intradural extramedullary components. This report aims to present the unusual imaging findings, discuss the potential differential diagnosis, and briefly highlight the work up and treatment for such a tumour.

## Clinical presentation

A 61-year-old female patient was evaluated in an outpatient setting for gradually worsening low back pain. Initially, the only other manifestation was right lower extremity pain but progressed to left leg pain that radiated to the patient’s left foot with perceived weakness. Additional symptoms included occasional urinary incontinence with Valsalva manoeuvres and occasional constipation. However, she denied overt bowel or bladder incontinence. The symptoms were described as constant and worse with standing. The patient was treated conservatively and sent for physical therapy and epidural steroid injections due to the clinical suspicion that symptoms were secondary to arthritic changes.

## Diagnosis and management

### Radiological examinations

**Figure 1.**
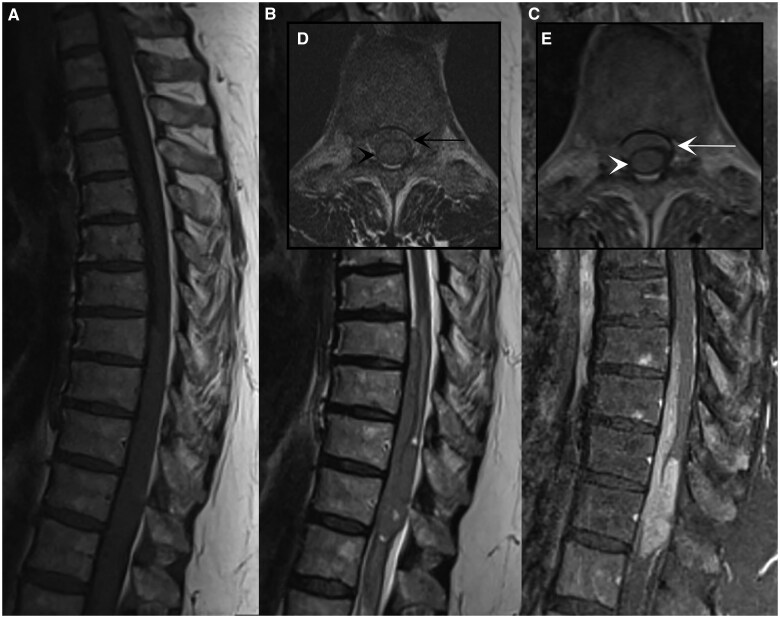
MRI of the thoracic spinal mass. (A) Sagittal T1-weighted: intramedullary and extramedullary mass is isointense to the spinal cord. (B and D) Sagittal and axial T2-weighted: mass appears mildly hyperintense to the cord with small cystic foci at the superior and inferior margins of the intramedullary component and associated cord oedema. On axial (D), there is cord expansion (black arrowhead) and a ventral extramedullary component (black arrow) which fills the thecal sac with no visible surrounding cerebral spinal fluid (CSF). (C and E) Sagittal T1-weighted fat saturation post-contrast and axial T1-weighted post-contrast: avid enhancement of both the intramedullary component (white arrowhead) and ventral extramedullary component (white arrow).

An MRI lumbar spine without contrast was ordered for continued back pain and left lumbar radiculopathy which revealed a partially visible intramedullary spinal cord mass in the conus expanding the cord with cord oedema. Subsequently, an MRI of the thoracic spine with and without contrast was obtained for further evaluation. This revealed a spinal cord mass measuring 9.6 × 1.4 × 1.4 cm (craniocaudal x anteroposterior x transverse) spanning the T8-T12 vertebral bodies. The mass contained an intramedullary component spanning approximately T10-T11 and a ventral intradural extramedullary component extending up to T8. The mass was isointense on T1-weighted imaging to the cord (Figure 1A), mildly hyperintense on T2-weighted imaging (Figure 1B and D) and showed avid, homogenous enhancement (Figure 1C and E). Additionally, extensive associated cord oedema was noted from T7 to the conus. There was no evidence for invasion into the neuroforamina or destructive bone changes. Full neuroaxis imaging was completed with MRI of the brain and remaining spine with and without contrast and no additional lesions were identified.

### Physical exam findings

At time of imaging, notable physical exam findings included: significant gait imbalance; mild weakness (4/5) in the right iliopsoas, knee flexors and extensors; diminished sensation in the bilateral distal lower extremities; and bilateral brisk patellar deep tendon reflexes. On additional neurological examination, she was classified as American Spinal Injury Association Impairment Scale (AIS) D at T3 on the right, and T11 on the left.

### Surgical findings, pathological results, and treatment

The patient underwent T7-T12 laminectomies with resection of spinal cord mass. Intraoperative ultrasound was used to confirm adequate exposure for the resection, and this also confirmed both intermedullary and extramedullary components of the tumour. The tumour was soft and brownish on visual inspection and the intramedullary component was significantly more vascular than the extramedullary component. The postoperative course was noneventful. Following the procedure, the patient underwent adjuvant radiotherapy. Twenty-one-day post-operative MRI showed successful resection of the enhancing mass with persistent cord oedema from T7-T10, improved from preoperative imaging.

Immunohistochemical stains included S-100, Sox-10, HMB-45, and Melan-A, which were all positive. IBRAF V600E immunostaining was negative, which reduced suspicion of metastasis from a cutaneous melanoma. Further testing with PET/CT and dermatological and ophthalmic examinations revealed no additional lesions suggestive of metastasis. The diagnosis was confirmed following expert consultation and specialized pathologic testing, including GNAQ p.Q209L missense variant. The histopathological examination returned with a diagnosis of primary melanoma of the spinal meninges.

## Discussion

Due to the rarity of primary spinal melanomas, sparse examples exist in the literature. Only 40 cases from a literature review in 2024 of primary melanoma in the spinal cord have been identified since 1906.^2^ Of these reports, approximately 42% of lesions are found in the thoracic segment.^2^ Spinal melanomas are more commonly intradural extramedullary (∼62%) but can also be intramedullary (∼38%) in location.^1^^,^^2^ Clinical symptoms are often non-specific, with pain being the most common complaint.^2^ Additionally, progressive weakness and asymmetric myelopathic symptoms may be noted due to compression of the spinal cord or spinal nerve roots.^2^

Understanding the development of melanocytes and their distribution in the body can help explain the varied locations of primary melanomas. During embryogenesis, melanoblasts arise from neural crest cells, which develop between the neural tube and ectoderm, and migrate to various locations throughout the body and mature into melanocytes, which are primarily found in the skin but are also present in the CNS, eyes, and mucous membranes.^1^^,^^3^ Within the CNS, melanocytes are primarily found in the leptomeninges and are the usual site of development for CNS malignant melanomas.^1^ Rarely, melanomas are seen in the intramedullary compartment. One theory to explain this is the failure of migration of neural crest cells during embryogenesis with these cells remaining with the neural tube which subsequently forms the spinal cord.^1^^,^^4^ Another possible explanation for atypical sites of CNS melanomas is from melanoblasts following the pia mater along vascular bundles.^1^

In our case, it is likely the tumour originated either from the leptomeninges, as observed in the Haider et al case,^2^ or originated in the cord, as observed in the Corrêa et al case,^1^ and subsequently grew into the other space. At the time of presentation, the extent of involvement of both spaces made it difficult to determine the site of origin. At surgery, there were 2 distinct components to the tumour with the extramedullary component appearing soft and brownish and the intramedullary component appearing more vascular, and it remained unclear surgically where the tumour originated. Pathology suggested a meningeal origin and helped confirm it was not a metastasis from a primary cutaneous melanoma.

MRI is the primary modality for assessing spinal cord tumours, but specific imaging features that conclusively identify melanoma are lacking, complicating differentiation from other spinal cord lesions. MRI presentation for non-cutaneous melanomas is classically thought to have hyperintense signals on T1-weighted imaging and iso- or hypointense signals on T2-weighted imaging.^1^^,^^3^^,^^5^ However, this is present in only 24%-47% of lesions and is related to the amount of melanin pigment and the presence of haemorrhage in the lesion.^3^^,^^5^ As is this case, amelanotic melanomas do not demonstrate the characteristic melanin appearance and will be isointense to hypointense on T1-weighted images.^3^ This emphasizes that the absence of T1-hyperintensity should not preclude melanoma from the differential diagnosis.

Spinal tumour differentials are usually organized by intramedullary, intradural extramedullary, or extradural location. However, when a tumour involves multiple spaces, it can be difficult to determine the site of origin and harder to build a differential diagnosis. There are limited examples in the literature of tumours that involve multiple spinal spaces. Given the intra and extramedullary location and signal characteristics in this case, the initial differential diagnosis was broad to include tumours of either location, including ependymoma or astrocytoma for a primarily intramedullary mass, and secondary considerations included lymphoma, metastatic disease, or solitary fibrous tumour of the dura.^6^^,^^7^ Some additional differential considerations include schwannoma,^8^ malignant peripheral nerve sheath tumour,^9^ and mucosa-associated lymphoid tissue lymphoma of the dura.^10^ Although this case did not show the classic hyperintense signal on T1-weighted images, if present, other pigmented lesions to consider include: metastatic malignant melanoma, meningeal melanocytoma, neurocutaneous melanosis, leptomeningeal melanomatosis, and melanotic schwannoma.^3–5^

For definitive diagnosis, histopathological examination is crucial and includes immunoreactivity tests for markers HMB-45, S-100 protein, and Melan-A.^5^ Primary CNS melanoma treatment primarily involves surgical resection, often supplemented with radiotherapy and, in some cases, chemotherapy. Differentiating primary from metastatic melanoma is essential due to significant prognostic implications. The prognosis for primary CNS melanoma tends to be more favourable. Regular surveillance imaging is recommended due to the unpredictable clinical course of the disease.^1^^,^^2^

This case highlights the complexity of diagnosing spinal melanomas, which often mimic more common spinal tumours such as ependymoma, astrocytoma, metastasis, or lymphoma. Often meningeal melanomas require extensive imaging and clinical evaluation to exclude other sites of potential primary melanoma. This underscores the challenges and the necessity of a comprehensive diagnostic approach often involving PET/CT, CT chest, abdomen, and pelvis, as well as skin and ophthalmologic examinations.^1^

## Conclusion

In summary, we have reported a rare case of a primary intramedullary and intradural extramedullary melanoma of the thoracic spine. Resection was achieved, and adjuvant radiotherapy was administered post-operatively. This case adds to the sparse literature by documenting a rare manifestation and could provide valuable insights into the diagnosis and management of similar cases.

## Learning points

- The classic hyperintense signal on T1-weighted MRI in non-cutaneous melanomas is present in only 24%-47% of lesions and is related to the amount of melanin pigment and the presence of haemorrhage in the lesion.
- Differential diagnostic considerations for a spinal tumour involving both the intermedullary and extramedullary spaces include but are not limited to: astrocytoma, ependymoma, primary spinal melanoma, metastatic disease, solitary fibrous tumour of the dura, schwannoma, malignant peripheral nerve sheath tumour, and mucosa-associated lymphoid tissue lymphoma of the dura.
- If primary CNS melanoma is within the differential, thorough radiological and clinical exams should be performed to exclude other sites of potential primary melanoma or other sites of metastases, including PET/CT, CT chest, abdomen, and pelvis, as well as skin and ophthalmologic examinations.
